# Community-Acquired Pneumonia and Empyema Caused by *Citrobacter koseri* in an Immunocompetent Patient

**DOI:** 10.1155/2015/670373

**Published:** 2015-11-08

**Authors:** Miguel Angel Ariza-Prota, Ana Pando-Sandoval, Marta García-Clemente, Ramón Fernández, Pere Casan

**Affiliations:** Hospital Universitario Central de Asturias (HUCA), Instituto Nacional de Silicosis (INS), Área del Pulmón, Facultad de Medicina, Universidad de Oviedo, 33011 Oviedo, Spain

## Abstract

*Citrobacter* species, belonging to the family Enterobacteriaceae, are environmental organisms commonly found in soil, water, and the intestinal tracts of animals and humans. *Citrobacter koseri* is known to be an uncommon but serious cause of both sporadic and epidemic septicemia and meningitis in neonates and young infants. Most cases reported have occurred in immunocompromised hosts. The infections caused by *Citrobacter* are difficult to treat with usual broad spectrum antibiotics owing to rapid generation of mutants and have been associated with high death rates in the past. We believe this is the first case described in the literature of a community-acquired pneumonia and empyema caused by *Citrobacter koseri* in an immunocompetent adult patient.

## 1. Introduction

The genus* Citrobacter* belongs to the family of* Enterobacteriaceae* and comprises 11 different species of facultative anaerobic, motile, Gram-negative bacilli, which are oxidase negative and typically utilize citrate as the sole carbon source [1]. Among* Citrobacter* species, the most commonly isolated from human clinical specimens are* C. koseri* (formerly named* C. diversus*),* C. freundii*,* C. youngae*,* C. braakii*, and* C. amalonaticus* [1].* Citrobacter* infections typically occur in hospital settings in patients with multiple comorbidities and seldom cause disease in the general population [2]. Neonates and immunocompromised hosts are highly susceptible to* Citrobacter* infections, which are mainly caused by* Citrobacter freundii* and* Citrobacter koseri*.* C. freundii* is usually associated with hepatobiliary tract infections, while* C. koseri* causes neonatal meningitis and brain abscess with high mortality rates [3].

In the environment,* Citrobacter* are commonly found in water, soil, and food and as occasional colonizers of the gastrointestinal tract of animals and humans [4]. Although* Citrobacter* strains colonizing the human gastrointestinal tract were traditionally considered to have low virulence [5], they can be the source of several types of infections [6], such as urinary tract, respiratory, intra-abdominal, wound, bone, bloodstream, and central nervous system infections [7–9]. We believe this is the first report of community-acquired pneumonia and empyema caused by* Citrobacter koseri* in an immunocompetent adult patient.

## 2. Case Presentation

A 72-year-old Spanish male was admitted to our hospital, 6 months ago, after two weeks of marked general syndrome (asthenia, hyporexia, and 3 Kg weight loss), accompanied with cough and mucopurulent sputum, moderate dyspnea, fever, night sweats, and right pleuritic chest pain. He had a 25-pack-year history of smoking and was diagnosed with arterial hypertension (HTN) in 2001. He worked as an architect and had no surgical background or other medical backgrounds of interest. He was taking Enalapril at the time.

The clinical findings were the following: body temperature 38°C; blood pressure 108/65 mmHg; heart rate 90 beats/min; respiratory rate 24 breaths/min; and oxygen saturation 93% (room air). The physical examination was normal, except for pulmonary auscultation, where diminished respiratory sounds and crackles were found bilaterally at the bases of both lungs. Laboratory tests revealed 24,800 × 10^9^ L white blood cell count with 88% neutrophils; 12.3 g/dL haemoglobin; the C-reactive protein (CRP) level that was 29 mg/L; procalcitonin (PCT) level 0.92 ng/mL; N-terminal probrain natriuretic peptide (NT-proBNP) level that was 400 pg/mL; glucose 129 mg/dL; and platelet count, arterial blood clotting, and the rest of biochemical tests that were within normal ranges. The arterial blood gases showed PaO_2_ 69 mmHg, PaCO_2_ 36 mmHg, pH 7.38, and standard HCO_3_ 37 mEq/L (room air).

**Figure 1:**
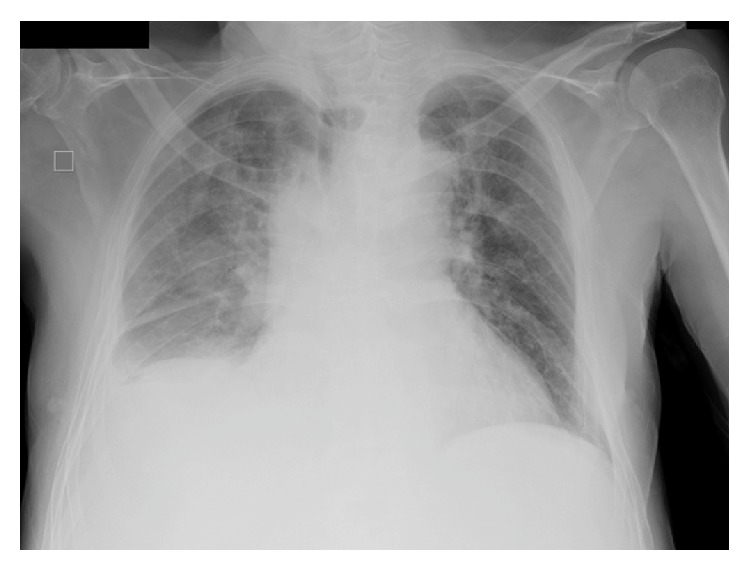
Chest X-ray on admission. Bilateral alveolar infiltrates with associated right pleural effusion.

**Figure 2:**
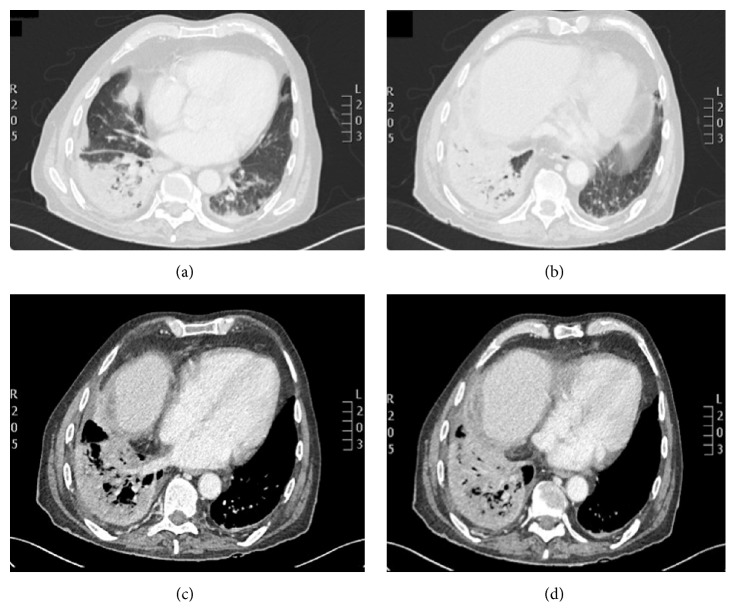
Computed tomography of the chest on admission. Right lower lobe alveolar consolidation with air bronchogram and in the left lower lobe and posterior segment of the left upper lobe similar lesions were identified in relation to a bilateral pneumonic process with associated loculated right pleural effusion and diffuse pleural thickening related to empyema.

**Figure 3:**
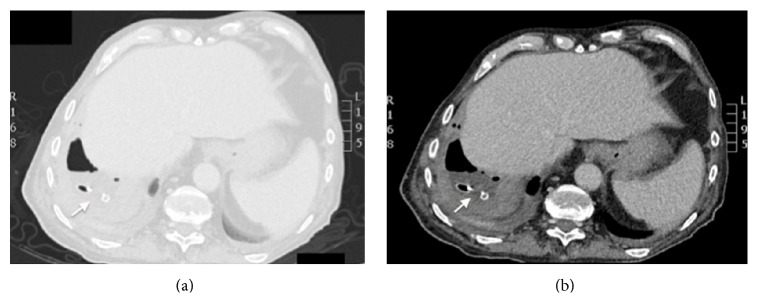
Computed tomography. Pigtail catheter correctly placed in the right lower lobe.

The chest X-ray revealed bilateral alveolar infiltrates with associated right pleural effusion (Figure 1). Urinary antigen for pneumococcus and* Legionella*, sputum cytology, mycobacterial culture, and serologic HIV tests were negative. Antibiotic treatment with piperacillin/tazobactam and levofloxacin was initiated on admission. A chest and abdomen computed tomography (CT) scan was performed two days after admission. The CT scan showed a right lower lobe alveolar consolidation with air bronchogram and in the left lower lobe and posterior segment of the left upper lobe similar lesions were identified in relation to a bilateral pneumonic process with associated loculated right pleural effusion and diffuse pleural thickening related to empyema (Figure 2). A subdiaphragmatic lesion was discarded. A diagnostic thoracocentesis was performed obtaining purulent fluid (empyema was confirmed). The pleural fluid biochemistry showed 430,000 white blood cells; 3000 red blood cells; glucose level 44 mg/dL; 22 g/L proteins; and pH of 6,99. A CT-guided pigtail catheter was correctly placed extracting 500 mL of purulent fluid (Figure 3). The patient showed clinical improvement with disappearance of the fever. The pleural fluid culture identified* Citrobacter koseri* and no other pathogen was isolated. The isolate was sensitive to amoxicillin clavulanic acid and piperacillin/tazobactam (resistant to ampicillin). The bacilloscopy, PCR* M. tuberculosis* (XPERT MTB/RIF), and mycobacterial cultures were negative.

**Figure 4:**
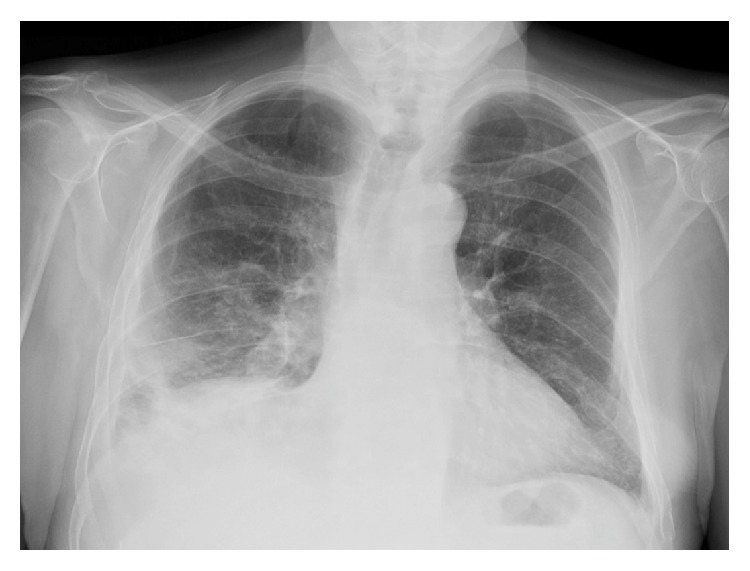
Control chest X-ray. Loss of volumen of the right lung and improvement of the right alveolar basal infiltrate.

After 12 days of intravenous antibiotic treatment, piperacillin/tazobactam and levofloxacin were suspended, and treatment with oral amoxicillin clavulanic acid (1000 mg/62,5 mg two tablets twice a day every 12 hours) was initiated with good tolerance and compliance. The patient was discharged with the diagnosis of bilateral pneumonia and right pleural empyema caused by* Citrobacter koseri*. In the October* follow-up* visit, the patient showed clinical improvement (residual dry cough, no fever, and decreased right chest pain) since he was discharged. The control chest X-ray showed loss of volumen of the right lung and improvement of the right alveolar basal infiltrate in comparison to the last X-ray performed during admission (Figure 4).

**Figure 5:**
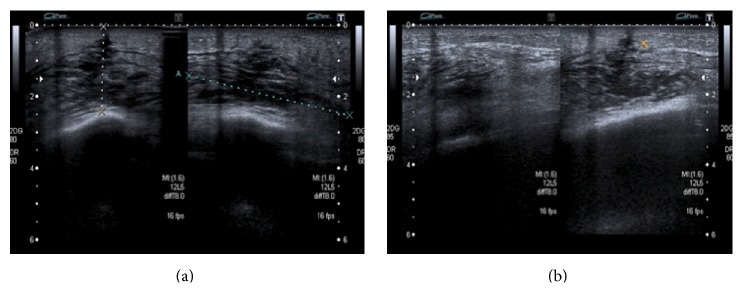
Right thoracic wall ecography. Fluid collection of 17 × 4 mm with a fistulous pleural tract with minimal pleural effusion (3.6 mm) associated with a small subcutaneous abscess in the area where the pigtail catheter was originally inserted, with the risk of producing a fistula to skin.

**Figure 6:**
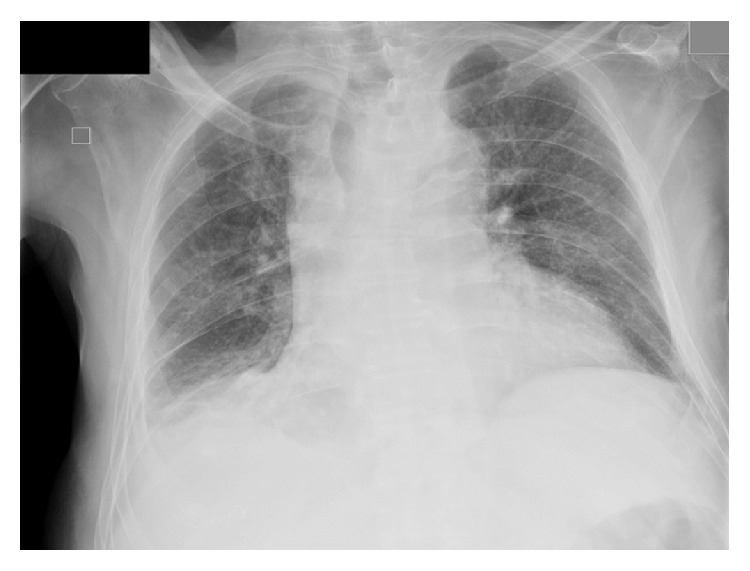
Control chest X-ray on the follow-up visit. Radiological improvement of the right lower lobe alveolar infiltrate.

**Figure 7:**
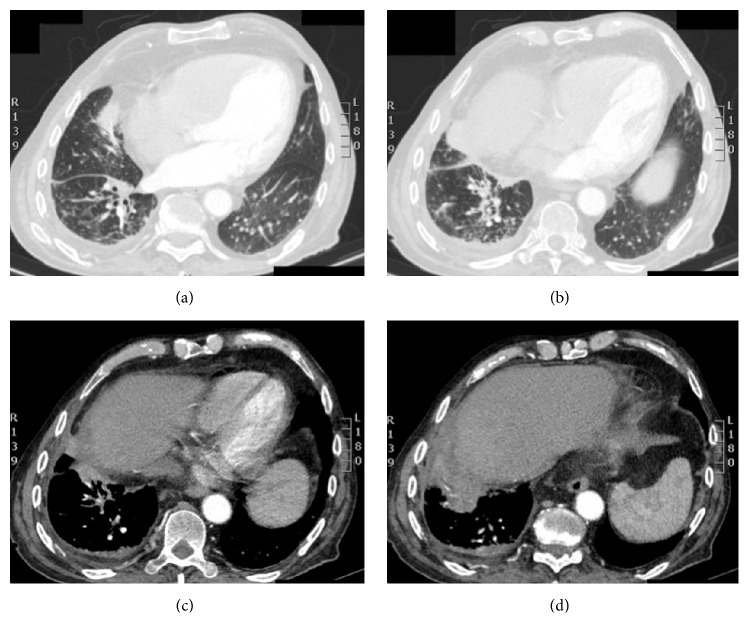
Control computed tomography of the chest. Almost complete resolution of the right lower lobe consolidation.

The patient was again admitted 2 weeks after discharge, because of swelling and pain in the area where the pigtail catheter was previously placed. An ecography of the right thoracic wall was performed. The ecography showed a fluid collection of 17 × 4 mm with a fistulous pleural tract with minimal pleural effusion (3.6 mm) associated with a small subcutaneous abscess in the area where the pigtail catheter was originally inserted, with the risk of producing a fistula to skin (Figure 5). The abscess was drained with a small incision on the skin, and the sample was sent to the microbiology department for culture. A new pigtail drainage catheter was placed, draining 200 mL of purulent fluid.* Citrobacter koseri* was isolated again in the area of the subcutaneous abscess and in the pigtail purulent fluid. The patient was discharged with amoxicillin clavulanic acid for one more month. A control chest X-ray performed four weeks later showed radiological improvement (Figure 6). In total, the patient was treated for 12 days with piperacillin/tazobactam and levofloxacin and for three months with amoxicillin clavulanic acid. A control CT scan performed 2 months ago showed almost complete resolution of the right lower lobe consolidation (Figure 7). The patient remained well on the 3-month* follow-up* visit.

## 3. Discussion

*Citrobacter*, a Gram-negative bacterium belonging to Enterobacteriaceae, is a rare cause of lung abscess.* Citrobacter* infections usually occur in patients with underlying comorbidities or immunosuppression [10]. The infections caused by* Citrobacter* are difficult to treat with usual broad spectrum antibiotics owing to rapid generation of mutants and have been associated with high death rates in the past [10]. In our case, the patient was an immunocompetent adult with no underlying important comorbidities, making this a very unusual clinical case because this organism commonly affects neonates and immunocompromised infants. A retrospective study from Taiwan on* Citrobacter* bacteraemia reported 45 patients over a period of thirteen years [10]. Patients with malignancies (48.9% mostly intra-abdominal) or hepatobiliary stones (22.2%) were found to have high predilection for* Citrobacter* bacteraemia. Abdominal cavity (51.1%) was the most common site for initial infection, with other sites being urinary tract (20%) and lung (11.1%) [10]. Intra-abdominal infections included hepatobiliary tree infection (including three patients who had liver abscesses), peritonitis, and perianal abscess [10]. Another report of three cases noted two patients one with* Citrobacter*-related iliopsoas abscess and another patient with renal and liver abscess in a patient with diabetes owing to* C. koseri* [11].

The literature on* Citrobacter* abscess in adults is scant [12]. We performed a PubMed search with the terms “*Citrobacter koseri*”, “*Citrobacter koseri* pneumonia”, and “*Citrobacter koseri* empyema”. Nine cases of abscess secondary to* C. koseri* infection in adults were found in this search. None of these cases were associated with lung abscess, pneumonia, or empyema. This is the first described case in the literature of community-acquired pneumonia and empyema caused by* Citrobacter koseri* in an immunocompetent adult patient. Regarding treatment, it was observed in the study on* Citrobacter*-related bacteraemia that use of a cephalosporin within 14 days promoted the emergence of cefotaxime-resistant strains and multidrug-resistant strains [10]. Another study at a north Indian tertiary institute depicted a high degree of resistance to the third-generation and the fourth-generation cephalosporins, as well as piperacillin, gentamicin, and ciprofloxacin [13]. In our case, the isolate was sensitive to piperacillin and all third and fourth generation cephalosporins. In spite of broad spectrum antibiotic treatment according to the sensitivity reports, patient's condition showed a very slow improvement;* Citrobacter koseri* was still isolated from the pigtail catheter drainage and subcutaneous abscess after 30 days of antibiotic treatment. Imipenem has been consistently found to be active against* Citrobacter* spp. [14, 15]. As for gentamicin, despite earlier reports showing the susceptibility of* Citrobacter* spp. to this agent [14], the rates of resistance appear to be rising [15]. Rising resistance to ciprofloxacin is also of concern [15]. We could speculate that a beta-lactamase inhibitor may become the first choice for complicated* Citrobacter* infection that requires prolonged courses of antibiotics.

## 4. Conclusion

The present case highlights* Citrobacter koseri* as a rare cause of empyema. Although* Citrobacter* infections occur more often in immunocompromised neonates and young infants predominantly causing meningitis and liver abscess, pneumonia and empyema should be added to the spectrum of disease in immunocompetent adult patients, where a combined and prolonged treatment (invasive intervention/drainage and medication) is probably the faster and more efficient solution.

## Abbreviations

HTN: Hypertension
CRP: C-reactive protein
PCT: Procalcitonin
NT-proBNP: N-terminal probrain natriuretic peptide
HIV: Human immunodeficiency virus
CT: Computed tomography.
